# Transgender Adolescent School Climate, Mental Health, and Adult Social Support

**DOI:** 10.1001/jamapediatrics.2024.3079

**Published:** 2024-08-26

**Authors:** Mollie T. McQuillan, Joseph R. Cimpian, Benjamin A. Lebovitz, Erin K. Gill

**Affiliations:** 1 University of Wisconsin–Madison, Madison; 2 New York University, New York

## Abstract

This cross-sectional study describes gender differences in adolescent school climate, health, and social supports, including who depressed or anxious youth seek help from.

Despite well-established health disparities between transgender and cisgender youth,^1^ state bills restricting gender-inclusive school supports and health care have dramatically expanded since 2019.^2^ While parental support for transgender youth strengthens healthy development,^3^ unsupportive parents contribute to suicidality and homelessness.^4^ This study examines (1) whether school climate and health gender inequities hold in Wisconsin, a politically contested state without nondiscrimination or bullying legislative protections for transgender youth, and (2) gender differences among adolescents who sought help when depressed or anxious.

## Methods

The 2021 Wisconsin Youth Risk Behavior Survey (WI-YRBS) collected data from September to December 2021 for 92 316 high school students with valid responses in our main outcomes of interest (eAppendix and eTables 1 and 2 in Supplement 1). We tested for statistically significant differences in transgender vs cisgender students’ health risks and support using Poisson and multinomial logistic regression, accounting for the complex, multistage sampling design with WI-YRBS–provided sampling weights and cluster robust standard errors. The relatively large cross-sectional sample of transgender youth (n = 3957) facilitated additional robustness checks with models that included covariates, a conservative transgender categorization, exclusion of potentially inaccurate observations,^5^ and stratification by race and ethnicity. We followed Strengthening the Reporting of Observational Studies in Epidemiology (STROBE) reporting guidelines and received approval from the University of Wisconsin–Madison and New York University institutional review boards.

## Results

### School Climate and Mental Health

Compared with cisgender students, transgender students reported higher risk of bullying (adjusted relative risk [aRR], 1.96; 95% CI, 1.84-2.08) (Table), skipping school because of feeling unsafe (aRR, 2.45; 95% CI, 2.18-2.76) and not belonging at school (aRR, 2.70; 95% CI, 2.51-2.90). Transgender students faced greater risk of anxiety (aRR, 1.54; 95% CI, 1.51-1.57) and reported about 2 to 3 times the risk of depression (aRR, 1.95; 95% CI, 1.89-2.01), self-harming behavior (aRR, 2.77; 95% CI 2.68-2.87), considering suicide (aRR, 2.89; 95% CI, 2.75-3.05), planning suicide (aRR; 3.01, 95% CI, 2.84-3.19), and attempting suicide (aRR, 3.29; 95% CI, 3.03-3.58) compared with cisgender students.

**Table.** Relative Risk Ratios for Transgender and Cisgender Adolescents’ School Climate, Mental Health, and Social Support

Factor	aRR (95% CI)		
	Raw/unadjusted model (n = 92 316)	Covariate-adjusted model (n = 92 316)^a^	Potentially invalid observations removed (n = 79 745)^b^
School climate			
Bullied	2.07 (1.95-2.20)^c^	1.96 (1.84-2.08)^c^	2.01 (1.87-2.16)^c^
Missed school for feeling unsafe	2.62 (2.31-2.96)^c^	2.45 (2.18-2.76)^c^	2.58 (2.23-2.98)^c^
Don’t belong at school	2.74 (2.54-2.95)^c^	2.70 (2.51-2.90)^c^	2.75 (2.51-3.01)^c^
Mental health			
Depressive symptoms	2.09 (2.02-2.15)^c^	1.95 (1.89-2.01)^c^	2.15 (2.07-2.23)^c^
Self-harmed	3.09 (2.98-3.20)^c^	2.77 (2.68-2.87)^c^	3.20 (3.07-3.33)^c^
Anxiety symptoms	1.65 (1.62-1.68)^c^	1.54 (1.51-1.57)^c^	1.68 (1.65-1.71)^c^
Considered suicide	3.12 (2.97-3.27)^c^	2.89 (2.75-3.05)^c^	3.25 (3.07-3.43^)^^c^
Planned suicide	3.23 (3.05-3.42)^c^	3.01 (2.84-3.19)^c^	3.25 (3.07-3.43)^c^
Attempted suicide	3.61 (3.33-3.91)^c^	3.29 (3.03-3.58)^c^	3.76 (3.41-4.13)^c^
Adult support			
≥1 Supportive adult at school	1.40 (1.32-1.48)^c^	1.39 (1.31-1.47)^c^	1.38 (1.29-1.48)^c^
Seek help from ≥5 adults	0.37 (0.34-0.42)^c^	0.39 (0.35-0.44)^c^	0.38 (0.34-0.43)^c^
Adult tries to meet needs	0.89 (0.87-0.91)^c^	0.89 (0.87-0.90)^c^	0.91 (0.89-0.93)^c^
Who students talk to when depressed or anxious	(n = 75 164)	(n = 75 164	(n = 59 510)
Able to get help	0.54 (0.50-0.60)^c^	0.54 (0.49-0.60)^c^	0.50 (0.45-0.56)^c^
Teacher or other school adult	1 [Reference]	1 [Reference]	1 [Reference]
Parent	0.26 (0.21-0.33)^c^	0.25 (0.20-0.32)^c^	0.26 (0.20-0.34)^c^
Other adult	1.16 (0.87-1.55)	1.13 (0.85-1.51)	1.26 (0.90-1.75)
Friend	0.75 (0.62-0.89)^d^	0.73 (0.61-0.87)^c^	0.78 (0.63-0.96)^d^
Sibling	0.52 (0.40-0.67)^c^	0.50 (0.39-0.67)^c^	0.57 (0.43-0.76)^c^
Not sure	0.70 (0.58-0.85)^c^	0.70 (0.58-0.85)^c^	0.72 (0.56-0.91)^c^

Abbreviation: aRR, adjusted relative risk.

^a^ See methods in the eAppendix in Supplement 1. The covariate-adjusted model accounted for student grade (9th, 10th, 11th, 12th), sex (female vs male), and race and ethnicity (Hispanic, non-Hispanic racially marginalized groups, and non-Hispanic White).

^b^ We identified and excluded potentially mischievous responders if adolescents reported 2 or more of the following: (1) no fruits in the last 7 days, (2) no water in the last 7 days, (3) weight in the top or bottom 3%, and (4) never seen a dentist (n = 12 571).

^c^ Statistically significant RR at *P* ≤ .001.

^d^ Statistically significant RR at *P* < .01.

### Adult Support

Transgender youth reported a greater likelihood of identifying at least 1 adult in school they could talk to compared with cisgender peers (aRR, 1.39; 95% CI, 1.31-1.47), but a lower likelihood of seeking help from 5 or more adults (aRR, 0.39; 95% CI, 0.35-0.44), identifying adults who helped with basic needs (aRR, 0.89; 95% CI, 0.87-0.90), or finding help when needed (aRR, 0.54; 95% CI, 0.49-0.60) compared with cisgender peers (Figure and Table). More cisgender youth reported not feeling depressed or anxious (18.6%) than transgender youth (1.4%). Among students who felt depressed or anxious (Figure and Table), transgender students sought help from parents (aRR, 0.25; 95% CI, 0.20-0.32), friends (aRR, 0.73; 95% CI, 0.61-0.87), and siblings (aRR, 0.50; 95% CI, 0.39-0.67) and reported uncertainty about who to seek help from (aRR, 0.70; 95% CI, 0.58-0.85) less than adults in schools when compared with cisgender students.

**Figure.**
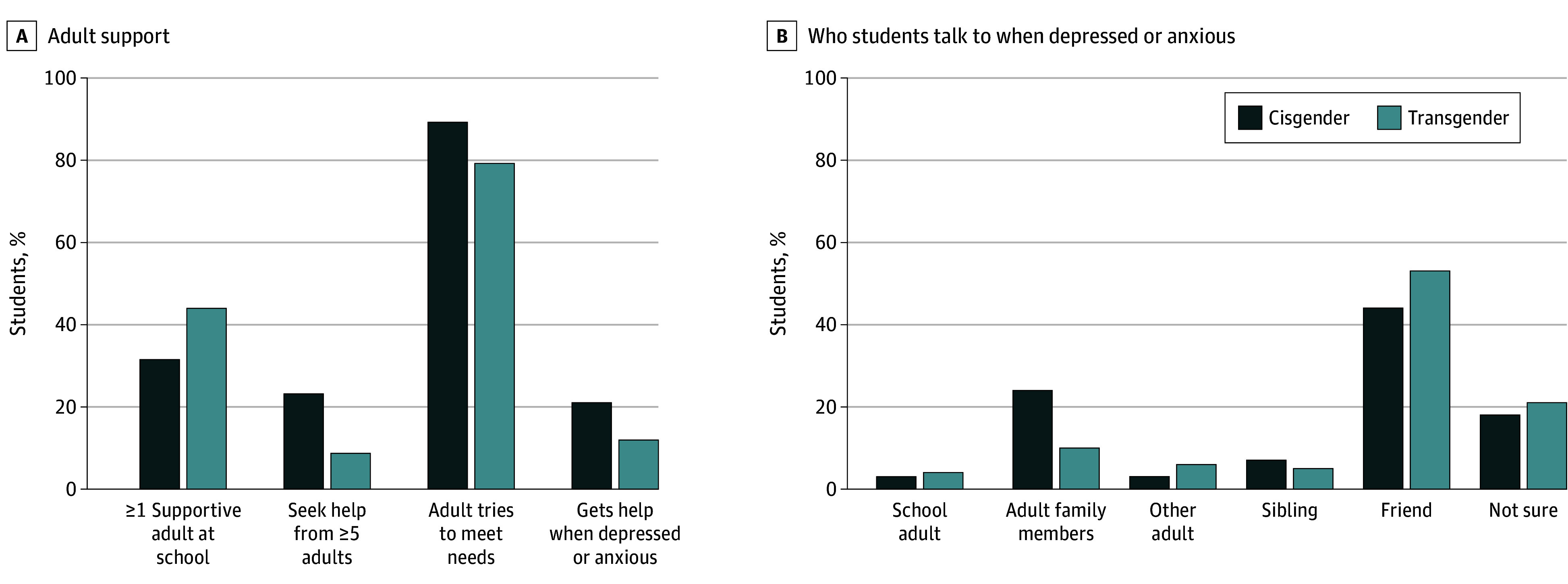
Social Support for Cisgender and Transgender Students as Reported in the 2021 Wisconsin Youth Risk Behavior Survey (N = 92 316) Sampling weights provided by the Wisconsin Department of Instruction were used to account for the complex sampling design of the survey. The frequencies are reported for adult social and emotional support (A) and who students talk to when depressed or anxious (B). The reported frequencies include students who said that (1) they had least 1 adult in school that they can talk to if having a problem, (2) aside from their parents, they have 5 or more adults they feel comfortable seeking support from if they have an important question in their life, (3) an adult in their life always or most of the time tries hard to meet their basic needs, and (4) they always or most of the time get the kind of help they need when they feel sad, empty, hopeless, angry, or anxious.

## Discussion

These novel findings suggest that schools serve as sites for both victimization and support for transgender youth. While YRBS design issues limit some inferences (eg, cross-sectional sample, exclusion of adolescents not enrolled in school), Wisconsin transgender students reported poor school climate and health concerns, which is consistent with past research^1^ and adds to a small but growing state-based literature. Importantly, we leverage the Wisconsin YRBS’s recent, unique adult social support items to reveal that adults in schools play a differentially greater role, and parents play a smaller role, when depressed or anxious transgender vs cisgender youth seek help. When situated in the literature discussing social supports as buffers to suicidality, depression, substance abuse, and other health risks,^1^ our study exposes the danger of restrictive education laws when school supports fill a critical need for transgender youth.

## Notes

Supplement 1. **eAppendix.** Methods of Youth Risk Behavior Survey
eReferences
**eTable 1.** Demographic Measures of YRBS Instrument **eTable 2.** Outcome Measures of YRBS Instrument

Supplement 2. Data sharing statement
