# Supplementary material for: Transgender Adolescent School Climate, Mental Health, and Adult Social Support
Source: JAMA Pediatr. 2024 Aug 26:e243079. Online ahead of print. doi: 10.1001/jamapediatrics.2024.3079 (PMC11348082; doi:10.1001/jamapediatrics.2024.3079)
Supplement: Supplement 1. — eAppendix. Methods of Youth Risk Behavior Survey eReferences eTable 1. Demographic Measures of YRBS Instrument eTable 2. Outcome Measures of YRBS Instrument [file jamapediatr-e243079-s001.pdf]

Supplemental Online Content

McQuillan MT, Cimpian JR, Lebovitz BA, Gill EK. Transgender adolescent school climate, mental health, and adult social support. *JAMA Pediatr*. Published online August 26, 2024. doi:10.1001/jamapediatrics.2024.3079

eAppendix. Methods of Youth Risk Behavior Survey
eReferences
eTable 1. Demographic Measures of YRBS Instrument
eTable 2. Outcome Measures of YRBS Instrument

This supplemental material has been provided by the authors to give readers additional information about their work.

eAppendix. Methods of Youth Risk Behavior Survey

DATA AND METHODS

The Centers for Disease Control and Prevention conducts the Youth Risk Behavior Survey (YRBS) biennially to assess the health behaviors of 9th through 12th-grade students in the United States. The 2021 Wisconsin YRBS (WI-YRBS) included 90 survey items and took place from September to December of 2021.[1] The YRBS cross-sectional survey design protects student privacy by allowing students to remain anonymous. The Wisconsin Department of Instruction requires schools notify parents and guardians that student participation is voluntary and follow informed consent procedures. The WI-YRBS provided post-stratification weights that account for the complex, multistage sampling design and nonresponse rates.

Participants. The 2021 WI-YRBS statewide sample included responses from 122,482 students across 369 high schools in 282 school districts in the state.[1] The 2021 statewide sample had a school response rate of 86%. This study's analytic sample includes respondents who indicated they identified as cisgender, transgender, or questioning their gender identity and provided valid responses on the main outcome measures used in the analysis (n=92,316). Respondents who indicated "I do not know what this question is asking" to the gender identity item were excluded from the analytic sample. Our cross-sectional study sample includes 95.7% cisgender, 4.3% transgender or questioning, 73.7% White, and 26.3% racially and ethnically marginalized groups (weighted frequencies). Most students identified as cisgender (75.8%), but 22.2% identified as LGBQ+, and 2% of students did not report a sexuality. Students categorized as female comprised 50.8% of the sample and male 49.2% of the sample. Participants came from 9th (25.7%), 10th (24.8%), 11th (25.2), and 12th Grade (24.3%; see eTable 1 for additional weighted frequencies of main demographic characteristics).

Analyses. We test for statistically significant differences in transgender versus cisgender students' school and health risks and social support using Poisson and multinomial logistic regression. We accounted for the complex, multistage sampling design with WI-YRBS–provided sampling weights and cluster robust standard errors in the analysis. All analyses were performed using Stata 18.

The research team performed several robustness checks, to test whether the statistically significant differences between transgender and cisgender students' reports of school climate, mental health, adult support, and whom students turn to when depressed or anxious held when using additional analytic approaches. The table in the article displays (1) the main analysis in addition to the robustness checks that accounted for (2) additional covariates of grade, race/ethnicity, and sex, and (3) excluded potentially invalid observations. Not included in the table were additional models that (4) accounted for additional covariates of grade, race/ethnicity, sex, and sexuality, (5) used a more conservative measure of transgender identity that excluded students questioning their gender, and (6) stratification by race/ethnicity.

Unadjusted Model. The main analysis used "I am not sure if I am transgender" and "Yes, I am transgender" to define the transgender group. The reference group was "No, I am not transgender." This categorization is used in all other models except for the "Conservative Trans Category" model, as discussed below.

© 2024 McQuillan MT et al. *JAMA Pediatrics*.

Covariate-Adjusted Model. We ran a covariate adjusted models to account for student reported grade (9th, 10th, 11th, 12th), sex (female vs. male), and race/ethnicity (Non-Hispanic White, Non-Hispanic Racially-Marginalized Groups, Hispanic). The research team created a categorical race/ethnicity variable. We categorized students as (1) Hispanic if they responded "yes" to "Are you Hispanic or Latino?," (2) Non-Hispanic White if they responded "no" to "Are you Hispanic or Latino?" and only selected "White" to "What is your race?," (3) Non-Hispanic Racially-Marginalized if they responded "no" to "Are you Hispanic or Latino?," and "American Indian or Alaska Native," "Asian," "Black or African American," or "Native Hawaiian or Other Pacific Islander" to "What is your race?." The reference groups included cisgender, 9th grade students, female assigned at birth, and White Non-Hispanic.

Excluding Potentially Invalid Responses. Past research indicates there could be measurement error in the YRBS identity items,[2] so the research team also assessed if our patterns remained robust if we excluded potentially invalid responders. Using the following items, we created an indicator variable to identify potentially mischievous responders: (1) no fruits in the last 7 days, (2) no water in the last 7 days, (3) weight in the top or bottom 3%, and (4) never seen a dentist. By excluding respondents who provided two or more responses that met these criteria of low incidence events, we account for potentially invalid observations (*n*=12,571).

Additional Robustness Checks. The research team also tested whether patterns held when using a more conservative categorization of transgender identity and when stratified by race. The conservative transgender identity variable only contained students who responded "Yes, I am transgender." While the main model includes students who responded that they may be questioning their identity with the "I am not sure if I am transgender" response, we exclude these "not sure" observations (*n*=2,098) in the more conservative model and then re-estimate the model.

The research team also tested if patterns held across racially- and ethnically-marginalized students and Non-Hispanic White students. We provide separate results for students reporting a Non-Hispanic White racial/ethnic identity (*n*=73,484) and for students of racially- or ethnically-marginalized groups who indicated any other marginalized racial or ethnic identity (*n*=18,832).

eReferences

1. Wisconsin Department of Public Instruction. 2023. "2021 Wisconsin Youth Risk Behavior Summary Report." https://dpi.wi.gov/sspw/yrbs.
2. Robinson-Cimpian JP. Inaccurate Estimation of Disparities Due to Mischievous Responders: Several Suggestions to Assess Conclusions. *Educ Res*. 2014;43(4):171-185. doi:10.3102/0013189X14534297

© 2024 McQuillan MT et al. *JAMA Pediatrics*.

eTable 1. Demographic Measures of YRBS Instrument

Variable	Survey Question	Response Options & Indicator Variable Construction			
Demographics		**Cisgender (0)**	**Transgender (1)**	**Excluded**	
Transgender – Liberal[a]	Some people describe themselves as transgender when their sex at birth does not match the way they think or feel about their gender. Are you transgender?	No, I am not transgender	Yes, I am transgender<br><br>I am not sure if I am transgender	I do not know what this question is asking	
Transgender – Conservative[b]	Some people describe themselves as transgender when their sex at birth does not match the way they think or feel about their gender. Are you transgender?	No, I am not transgender	Yes, I am transgender	I do not know what this question is asking<br>I am not sure if I am transgender	
Grade (individual indicator variables)	In what grade are you?	**9th (0)**	**10th (1)**	**11th (1)**	**12th (1)**
		**Non-Hispanic White (0)**	**Non-Hispanic Racially-Marginalized Groups (1)**	**Hispanic (1)**	
Race /Ethnicity (individual indicator variables)	Are you Hispanic or Latino?<br><br>What is your race? (Select one or more responses.)	No<br><br>White only	No<br><br>American Indian or Alaska Native<br>Asian<br>Black or African American<br>Native Hawaiian or Other Pacific Islander	Yes	
Sex	What is your sex?	**Female (0)**	**Male (1)**		

Notes: [a] Liberal transgender variable used in main analyses. [b] Conservative transgender variable used as a robustness check.

© 2024 McQuillan MT et al. *JAMA Pediatrics*.

eTable 2. School Climate, Mental Health, and Social Support Measures of YRBS Instrument

Variable	Survey Question	Response Options & Indicator Variable Construction	
School Climate		**0**	**1**
Bullied	During the past 12 months, have you ever been bullied on school property?	No	Yes
Skip School because Felt Unsafe	During the past 30 days, on how many days did you not go to school because you felt you would be unsafe at school or on your way to or from school?	0 days	1 day<br>2 or 3 days<br>4 or 5 days<br>6 or more days
Belonging at School	Do you agree or disagree that you feel like you belong at your school?	Strongly Agree<br>Agree<br>Not Sure	Strongly Disagree<br>Disagree
Mental Health		**0**	**1**
Depressive Symptoms	During the past 12 months, did you ever feel so sad or hopeless almost every day for two weeks or more in a row that you stopped doing some usual activities?	No	Yes
Self-Harmed	During the past 12 months, how many times did you do something to purposely hurt yourself without wanting to die, such as cutting or burning yourself on purpose?	0 times	1 time<br>2 or 3 times<br>4 or 5 times<br>6 or more times
Anxiety Symptoms	During the past 12 months, have you had significant problems with feeling very anxious, nervous, tense, scared, or like something bad was going to happen?	No	Yes
Considered Suicide	During the past 12 months, did you ever seriously consider attempting suicide?	No	Yes
Planned Suicide	During the past 12 months, did you make a plan to attempt suicide?	No	Yes
Attempted Suicide	During the past 12 months, did you ever attempt suicide?	0 times	1 time<br>2 or 3 times<br>4 or 5 times<br>6 or more times
Adult Support		**0**	**1**
1+ Supportive Adult at School	Is there at least one teacher or other adult in your school that you can talk to if you have a problem?	No<br>Not Sure	Yes

© 2024 McQuillan MT et al. *JAMA Pediatrics*.

Seek Help from 5+ Adults	Besides your parents, how many adults would you feel comfortable seeking help from if you had an important question affecting your life?	0 adults<br>1 adult<br>2 adults<br>3 adults<br>4 adults	5 or more adults
Adult Tries to Meet Needs	During your life, how often has there been an adult in your household who tried hard to make sure your basic needs were met, such as looking after your safety and making sure you had clean clothes and enough to eat?	Never<br>Rarely<br>Sometimes	Most of the time<br>Always
Gets Help when Depressed or Anxious [a]	When you feel sad, empty, hopeless, angry, or anxious, how often do you get the kind of help you need?	Never<br>Rarely<br>Sometimes	Most of the time<br>Always
Who Students Talk to When Depressed or Anxious			
Who Students Talk to When Depressed or Anxious [a]	When you feel sad, empty, hopeless, angry, or anxious, with whom would you most likely talk about it?	Parent or another adult family member<br>Teacher or other adult in this school (reference group)<br>Other adult<br>Friend<br>Sibling<br>Not sure	

Notes: [a] Analysis tested patterns of students who reported they felt depressed or anxious and excluded 17,152 participants who responded “I do not feel sad, empty, hopeless, angry, or anxious.”

© 2024 McQuillan MT et al. *JAMA Pediatrics*.
