# Supplementary material for: Transgender Adolescent School Climate, Mental Health, and Adult Social Support
Source: JAMA Pediatr. 2024 Aug 26:e243079. Online ahead of print. doi: 10.1001/jamapediatrics.2024.3079 (PMC11348082; doi:10.1001/jamapediatrics.2024.3079)
Supplement: Supplement 2. — Data sharing statement [file jamapediatr-e243079-s002.pdf]

Data Sharing Statement

McQuillan. Transgender Adolescent School Climate, Mental Health, and Adult Social Support. *JAMA Pediatr*. Published August 26, 2024. doi:10.1001/jamapediatrics.2024.3079

Data
Data available: No

Additional Information
Explanation for why data not available: The data is protected due to privacy and confidentiality issues. We have access through a data use agreement with the WI Department of Instruction
